# Removing Batch Effects in Analysis of Expression Microarray Data: An Evaluation of Six Batch Adjustment Methods

**DOI:** 10.1371/journal.pone.0017238

**Published:** 2011-02-28

**Authors:** Chao Chen, Kay Grennan, Judith Badner, Dandan Zhang, Elliot Gershon, Li Jin, Chunyu Liu

**Affiliations:** 1 National Ministry of Education Key Laboratory of Contemporary Anthropology, Fudan University, Shanghai, People's Republic of China; 2 Department of Psychiatry, University of Chicago, Chicago, Illinois, United States of America; 3 Department of Pathology, Zhejiang University, Hangzhou, People's Republic of China; University of California, Davis, United States of America

## Abstract

The expression microarray is a frequently used approach to study gene expression on a genome-wide scale. However, the data produced by the thousands of microarray studies published annually are confounded by “batch effects,” the systematic error introduced when samples are processed in multiple batches. Although batch effects can be reduced by careful experimental design, they cannot be eliminated unless the whole study is done in a single batch. A number of programs are now available to adjust microarray data for batch effects prior to analysis. We systematically evaluated six of these programs using multiple measures of precision, accuracy and overall performance. ComBat, an Empirical Bayes method, outperformed the other five programs by most metrics. We also showed that it is essential to standardize expression data at the probe level when testing for correlation of expression profiles, due to a sizeable probe effect in microarray data that can inflate the correlation among replicates and unrelated samples.

## Introduction

Gene expression microarray technology [1], [2], [3], [4] measures the expression of thousands of genes in a single assay, using multiple probes to assay each transcript. It is a revolutionary tool for identifying genes or pathways whose expression changes in response to specific perturbations. Promising as it is, there are concerns regarding the reliability, and hence the utility, of DNA microarray technology in the study of physiological processes and diseases [5], [6].

Gene expression microarray results can be affected by minuscule differences in any number of non-biological variables[7], so reagents from different lots, different technicians or even changing atmospheric ozone levels[8] can impact the data. Here, the term “batch” refers to microarrays processed at one site over a short period of time using the same platform. The cumulative error introduced by these time and place-dependent experimental variations is referred to as “batch effects."

Batch effects are almost inevitable; largely because most of the available microarray platforms can assay fewer than 24 samples at a time (the latest technology may process 96 samples in each batch). Since hundreds or thousands of samples may be needed for population studies, samples for high-throughput microarray studies must often be processed at different times and/or sites. However, of the thousands of DNA microarray papers published every year, few address the problem. Of the 219 papers using microarray data published from January 1 to July 1, 2010, less than ten percent addressed this issue (NCBI GEO database, studies with more than 30 samples)[9].

A number of approaches have been used or developed for identifying and removing batch effects from microarray data[10], of which we have chosen six for evaluation. Distance-weighted discrimination (DWD)[11], based on the Support Vector Machines (SVM) algorithm, is a two-class discrimination analysis for high-dimension low sample size data. Mean-centering (PAMR)[12] is a gene-wise one-way analysis of variance (ANOVA). Surrogate variable analysis (SVA)[13], combines singular value decomposition (SVD) and a linear model analysis to estimate the eigenvalues from a residual expression matrix from which biological variation has already been removed. Geometric ratio-based method (Ratio_G) scales sample measurements by the geometric mean of a group of reference measurements [14]. An Empirical Bayes method, called Combating Batch Effects When Combining Batches of Gene Expression Microarray Data (ComBat)[15], estimates parameters for location and scale adjustment of each batch for each gene independently[16]; ComBat includes two methods, a parametric prior method (ComBat_p) and a non-parametric method (ComBat_n), based on the prior distributions of the estimated parameters. We excluded the following algorithms either because they have already been shown by previous studies to be inferior to one or more of the methods we are analyzing, or because they are minor variations of those methods[13], [14], [15]: singular value decomposition (SVD)[17], standardization (Location/Scale adjustment model)[16], a ratio-based method with arithmetic mean (Ratio_A)[14]. Sources for the programs we evaluated, plus some of their computational features, are provided in Table S1.

Ideally, all these batch adjustment methods would produce comparable results; however, since they are based on different statistical models, their accuracy, precision and overall effectiveness vary. In this paper, we sought to identify the algorithm that removes batch effects most effectively, including striking the optimal balance between precision and accuracy. Simulated data were used for each initial assessment, so the true values would be known *a priori*, and experimental data were used for verification.

We created two simulation data sets. The first was the Variation Assessment Simulated (VAS) data set, comprising 100 samples, 65 of which were assigned to Profile 1 and 35 of which were assigned to Profile 2, where profile was a generic random variable. Expression values for 991genes were simulated, all of them differentially expressed between the two profiles. This data set was generated twice, with different levels of batch effects incorporated each time, creating technical duplicates for comparison. The second simulated set, the Accuracy Assessment Simulated (AAS) data, consisted of 100 cases and 100 controls, with 1,200 out of 10,000 genes being differentially expressed with 12 different fold change values ranging from −3 to 3. The data incorporated a range of batch sizes and variable amounts of batch effects.

Both experimental brain expression data sets were produced using the Affymetrix GeneChip Human Genome U133A Array, a short oligonucleotide cDNA microarray. The Stanley Medical Research Institute (SMRI) data set included three technical replicates of 62 individuals, with each replicate processed in one of three laboratories [18] Based on place and date of processing, the samples were run in 23 batches, averaging eight samples with at least one case and one control per batch. The Affymetrix U133A spike-in set (http://www.affymetrix.com/support/technical/sample_data/datasets.affx), comprised three technical replicates of 14 separate hybridizations of 42 spiked transcripts at concentrations ranging from 512 pM down to 0.125 pM. Like the simulation data, true positives and true negatives were known for this data set.

The VAS and SMRI data were used for variation and precision assessment; the AAS and spike-in data were used for accuracy and overall performance evaluation. All the batch adjustment methods were applied after experimental data were pre-processed by robust multiarray analysis (RMA)[19], which summarizes the probe level expression data into a probe set level expression value; a probe set consists of 11 to 20 probes used to assay the expression of one gene or exon. The simulated data sets were defined as the probe set level expression values that would be obtained from RMA processing.

We first measured how much each program reduces the variation caused by batch effects. In the VAS data set, the variation came only from batch and Profile effects. To verify our simulation result, we used SMRI data, in which nine factors are considered possible sources of variation and batch effects were divided into site and date effects separately. In both the simulated data and experimental data, variation attributable to batch effects before and after batch adjustment were identified using principal variation component analysis (PVCA)[10], [20].

To test the programs' precision, we assessed whether the expression values of the technical replicates correlated better before or after batch adjustment, first between the two replicates from the VAS data using Pearson's correlation coefficient, then among the three SMRI replicates as assessed by intraclass correlation (ICC).

As a measure of accuracy, the programs' abilities to accurately quantify fold change in expression were assessed using the correlation between nominal fold changes and observed fold changes. The *signal detection slope* [21], i.e., the slope of the line produced by regressing the nominal fold change against the observed fold change, was also calculated. The Affymetrix spike-in dataset was used to verify the result.

To assess the overall detection ability of each program, we used a receiver operator characteristic (ROC) curve. ROC curves plot the true positive rate (i.e., sensitivity) against the false positive rate (i.e., 1-specificity). The actual test statistic is the area under the curve (AUC); the program with the optimal combination of sensitivity and specificity will have the largest area of AUC. The ROC-AUC of the AAS data was calculated, and then the Affymetrix spike-in data was used to validate the simulation result. We also tested each program's ROC-AUC result with variously sized batches in the AAS data.

Our ultimate goal was to identify the batch adjustment method that best prepares data from multiple batches for analysis or meta-analysis to be integrated, as measured by batch effects reduction, accuracy, precision and overall performance.

## Results

### Proportion of variation attributable to batch effects

We used principal variation component analysis (PVCA)[10], [20] to measure how much variation in the expression data was attributable to batch effects. PVCA estimates source and proportion of variation in two steps, principal component analysis (PCA) and variance component analysis (VCA). The principal components (PCs) identified in the PCA that together account for a predetermined proportion of variation, here 60%, are retained for the VCA. The VCA uses a linear model to match each PC to a known source of variation, in this case batch effects, profile effects and interaction between batch and profile effects. The variation in each PC is weighted by its eigenvalue from PCA, and the resulting value represents the overall variation explained by that component.

**Figure 1:**
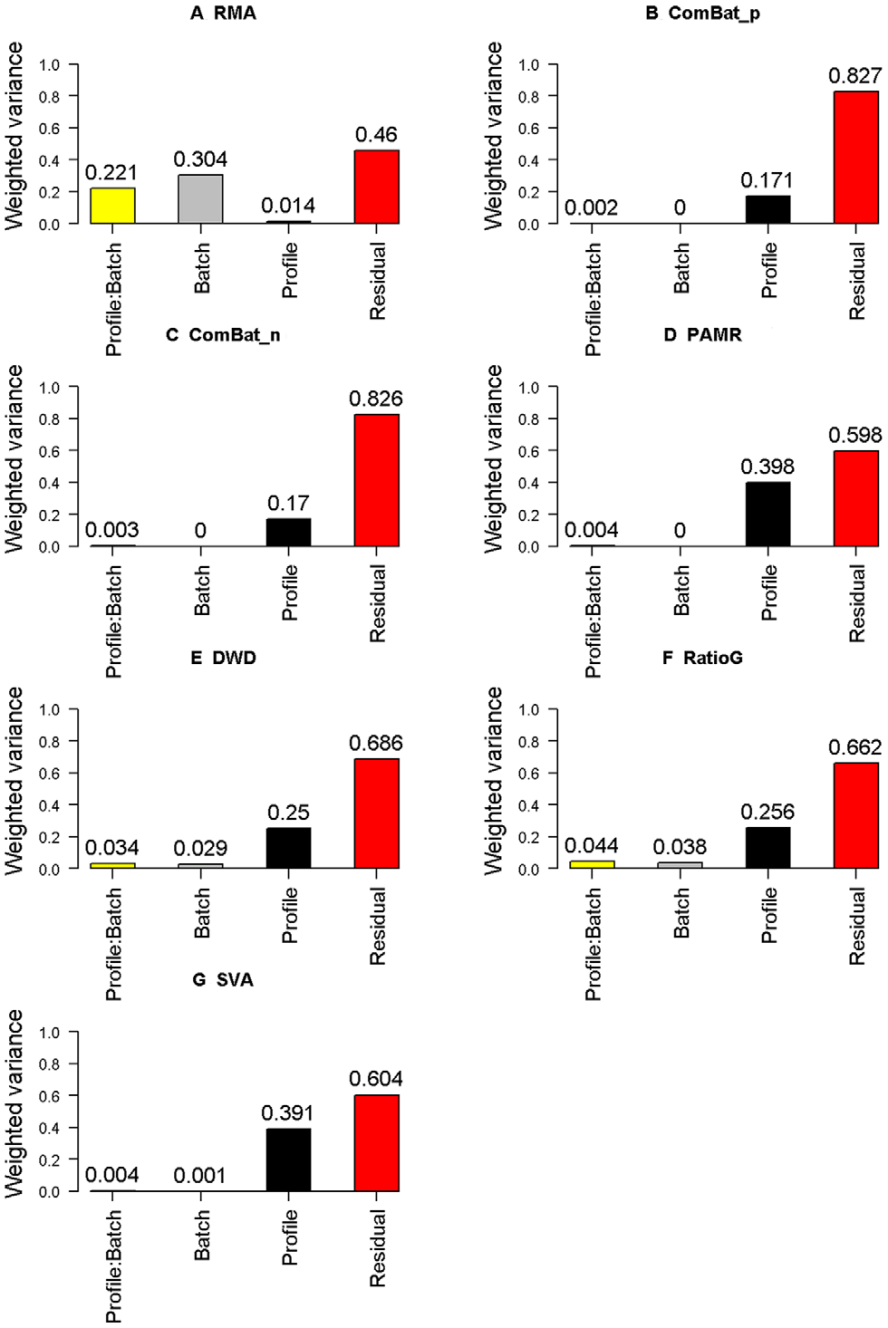
PVCA results in VAS data. The contribution of each factor to the overall variation was estimated by PVCA. All the effects, including batch effects, Profile effects, interaction between batch and Profile effects, and residuals, were estimated for their contribution to the overall variation. A. Data without batch adjustment. B. Data processed by ComBat_p as batch adjustment tool/model. C. Data processed by ComBat_n. D. Data processed by PAMR. E. Data processed by DWD. F. Data processed by Ratio_G. G. Data processed by SVA.

The PVCA revealed that batch effects explained 30.4% of the overall variation in the VAS RMA data (Figure 1A). All batch adjustment methods reduced that variation to some degree, and three, ComBat_p, ComBat_n and PAMR, eliminated it completely (**Figure 1B**). Interaction between batch and profile explained 22.1% of the variation in the RMA data, which was reduced to less than 5% by all batch adjustment methods and to less than 1% by ComBat_p, ComBat_n, PAMR and SVA. This reduction made the biological variation due to profile more apparent, increasing it from 0.014 (RMA) to 0.171(ComBat_p), 0.170 (ComBat_n), 0.398 (PAMR), 0.250 (DWD), 0.391 (SVA) and 0.256 (Ratio_G) after the removal of batch effects.

**Figure 2:**
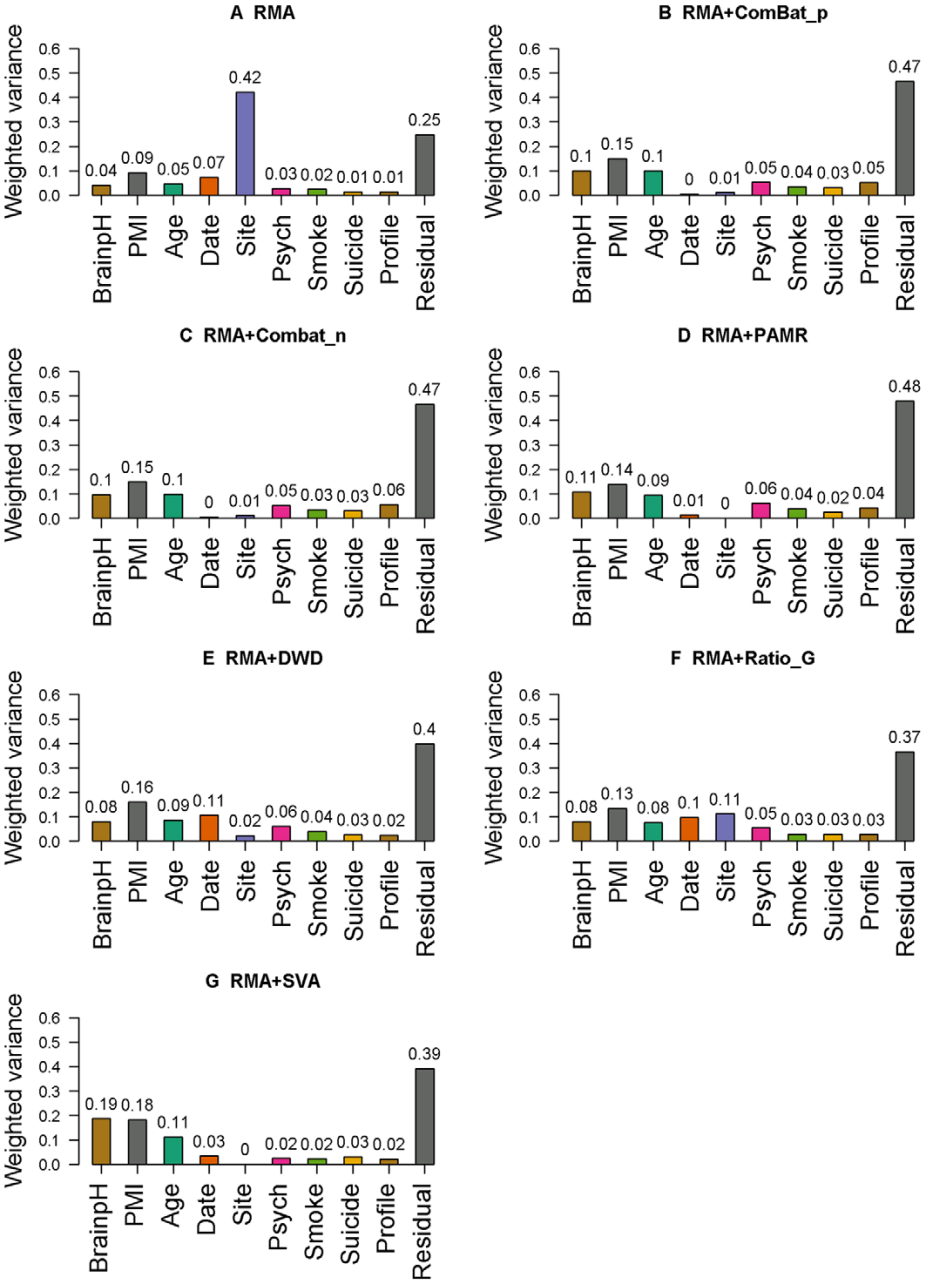
PVCA results in SMRI data. The contribution of each factor to the overall variation was estimated by PVCA, A. Data without batch adjustment. B. Data preprocessed by RMA with ComBat_n as batch adjustment tool/model. C. Data preprocessed by RMA with ComBat_p. D. Data preprocessed by RMA with PAMR. E. Data preprocessed by RMA with DWD. F. Data preprocessed by RMA with Ratio_G. G. Data preprocessed by RMA with SVA.

We obtained similar results from the experimental SMRI data set (Figure 2). This time, we considered nine possible sources of variation, as provided by SMRI: date effects, site effects, disease profile, brain pH, post-mortem interval (PMI), age, suicide status, smoking status at time of death and presence of psychotic features. The first seven PCs met our 60% threshold of variation. In the unadjusted data, PVCA showed that batch effects were responsible for almost half of the overall variation detected by the first seven PCs, including 42% attributable to site effects and 7% to date effects **(** **Figure 2A** **)**. The next largest effect came from PMI, which accounted for only 9% of the variation.

After applying ComBat_p or ComBat_n, only 1% of the total variation was still attributable to batch effects: this represents a 98% reduction in batch effects and a 48% reduction in total variation. PAMR was almost as effective, reducing batch effects by 98%. SVA, DWD and Ratio_G were less so, at 94%, 73% and 57%, respectively.

### Precision

Precision refers to the closeness of the set of values obtained from multiple testing of identical samples. The precision of expression measures can be assessed by testing correlation among replicates, which we did between the VAS duplicates using Pearson's correlation coefficient and among the 62 SMRI triplicates using intraclass correlation coefficient (ICC) [22]; ICC is appropriate for assessing correlation among groups, while Pearson's is intended to measure correlation between pairs.

First, however, due to the sizeable probe effect in microarray data, which can inflate the correlations between sample pairs regardless of whether the sample pairs are replicates[23], we standardized each probe set value to a mean expression value of 0 and a standard deviation (s.d.) of 1. Prior to standardization, the correlations between both replicate and non-replicate samples were all >0.9; afterwards, the median correlation among non-replicates was zero (**Figure S1**).

After this standardization, we calculated Pearson's correlation coefficient of the replicates in VAS data, before and after batch adjustment. To compare the difference of the correlation coefficients, we transformed the correlation r to approximately normally distributed z-scores (**Figure S2**). All batch removal methods increased the replicates' correlation: the improvements of the probes' correlation distribution were all significant with p<0.0001. ComBat and DWD showed the largest median differences (**Table S3, Row 6**).

**Figure 3:**
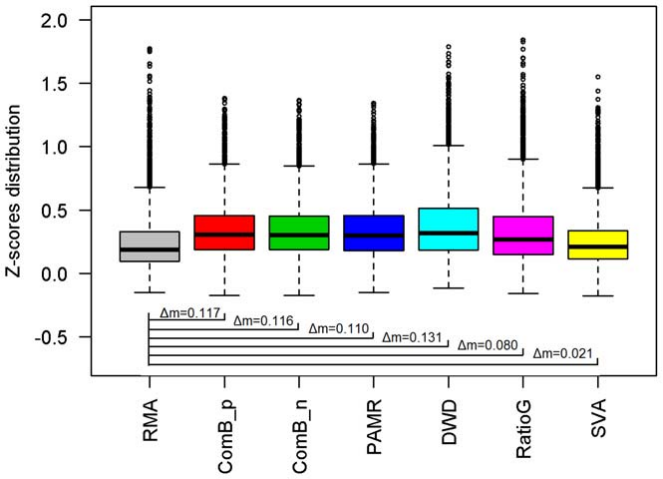
Distribution of SMRI ICCs after transformation. Boxplots of the distribution of z-scores transformed from intraclass correlation coefficients of probe set expression levels between three SMRI technical replicates. The methods are listed along the X axis. The Y axis is the distributions of all probe sets' ICC z-scores. The top of the box represents the top of the third quartile, the bottom of the box represents the bottom of the first quartile, the middle bar is the median value, box whiskers extend to 1.5 times the interquartile range from the box and circles are possible outliers. Δmedian indicates the median difference of z-score distributions between RMA data and data that has been processed with both RMA and the batch-adjustment method. Except for SVA, all batch adjustment methods significantly increased z-scores (p<0.0001).

In the SMRI experimental data, ICC was calculated for each probe set among the three replicate groups. The median z-score of the raw data was 0.188; after batch correction, the median z-scores were 0.305 for ComBat_p, 0.304 for ComBat_n, 0.298 for PAMR, 0.319 for DWD and 0.268 for Ratio_G. The increases in median ICC z-scores were all significant (p-values<0.0001). The differences among ComBat_n, ComBat_p, PAMR and DWD were not significant, but they were all significantly better than Ratio_G (p<0.0001). Only SVA failed to increase the median z-score significantly (0.209, p = 0.407) (**Figure 3** **; Table S3, Row 7**).

### Accuracy

**Figure 4:**
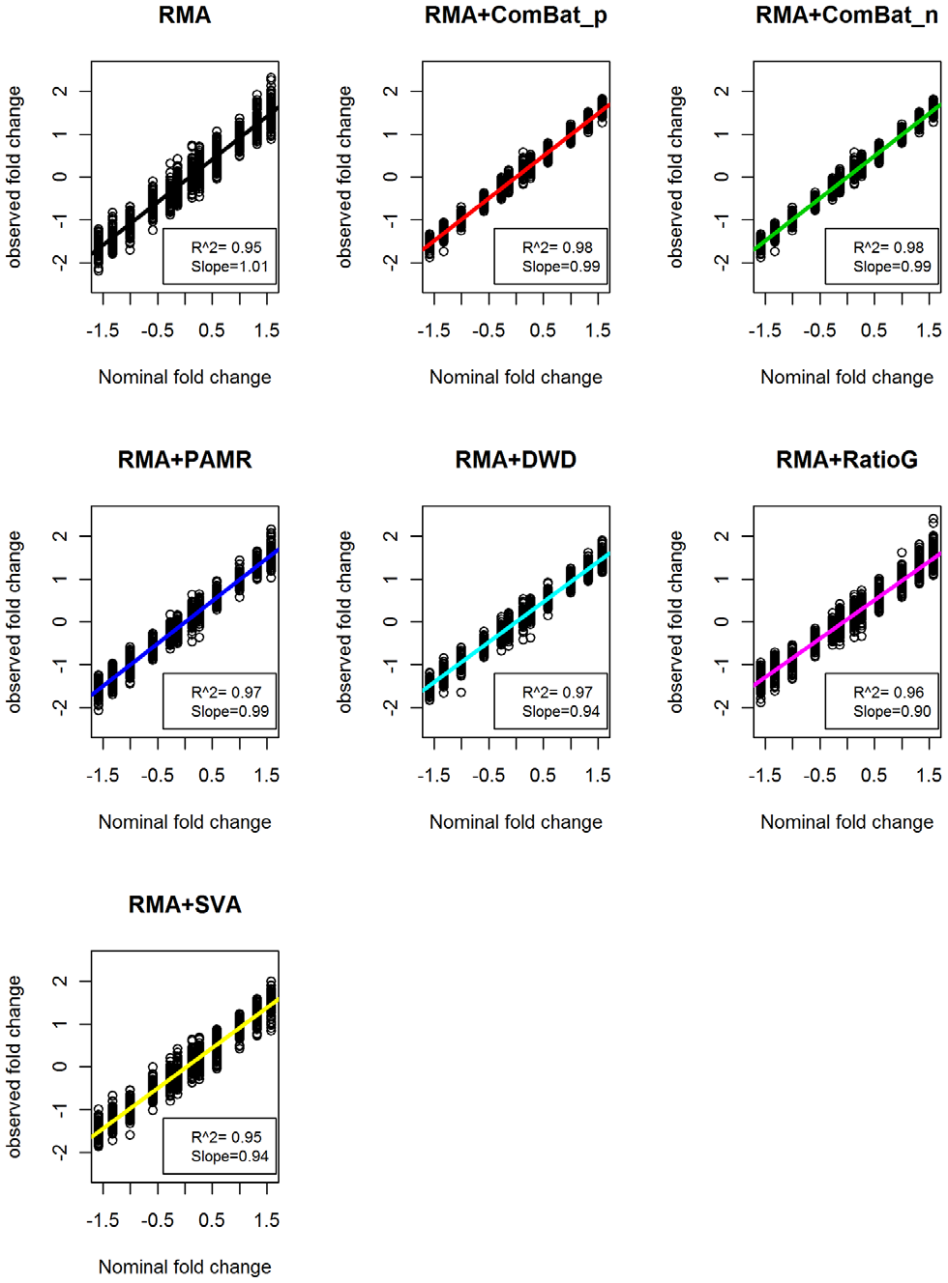
Correlation between the nominal fold changes and observed fold changes in AAS data. Correlation between the nominal fold changes and observed fold changes in RMA data and data after batch adjustment programs. We simulated 1200 genes out of 10000 genes as differentially expressed, with log 2 fold change range −1.58, −1.32, −1, −0.58, −0.26, −0.14 and 0.14, 0.26, 0.58, 1, 1.32, 1.58, responding to fold changes that range −3, −2.5, −2, −1.5, −1.2, −1.1 and 1.1,1.2,1.5,2,2.5,3 to reflect the approximate number of differentially expressed genes in the real data. The regression slopes were shown in colors by different program. Correlation coefficients (r^2^) were shown in legend, separately.

Accuracy refers to the closeness of a single measurement to its true value. We assessed accuracy by calculated how much each program increased the correlation between nominal fold changes and the observed fold changes in the AAS data after the fold changes were transformed to log 2 scale. Except for SVA (Spearman's correlation, r^2^ = 0.95), all programs increased the correlation between nominal log 2 fold change value and observed fold change value (p<0.0001), ComBat_p (r^2^ = 0.98), ComBat_n (r^2^ = 0.98), PAMR (r^2^ = 0.97), DWD (r^2^ = 0.97), Ratio_G (r^2^ = 0.96), compared to the unadjusted RMA result (r^2^ = 0.95) (**Figure 4**). Although small, the differences in correlation coefficient between ComBat (both ComBat_n and ComBat_p) and the next best program, DWD, were also significant (p<0.0001).

We also calculated each program's *signal detection slope* [21], which is the slope of the line produced by regressing nominal fold change against observed fold change; a slope of 1 indicates that nominal fold changes and observed fold changes are identical. The signal detection slope of the 1,200 nominally differentially-expressed genes in our unadjusted simulated data set was 1.01. There was no room for improvement, and none of the methods improve upon the RMA result; SVA, Ratio_G and DWD actually significantly decreased the accuracy, with Ratio_G decidedly the worst with the significantly lower slope of 0.90 (p<0.0001) (**Table S3, Row 10**).

To verify these results, we measured each program's correlation and slope in the Affymetrix experimental spike-in data set. SVA had the worst correlation again (r∧2 = 0.56 vs. r∧2 = 0.90, RMA data, p<0.0001), and decreased the slope from 0.68 to 0.51 (p<0.0001) **(Figure S3)**.

### Overall performance

**Figure 5:**
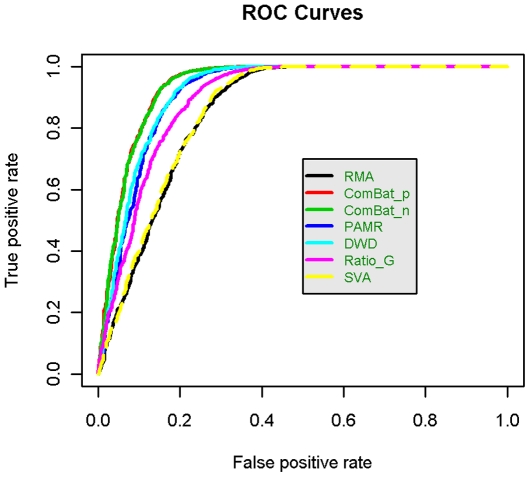
ROC curves in AAS data. ROC curves are graphical representations of both specificity and sensitivity that take into account both differentially and non-differentially expressed genes. ComBat_p and ComBat_n performed almost identically, so their curves overlap each other almost completely.

We used ROC curves to determine which program best optimized both sensitivity and specificity, i.e., maximized true positives (TP) while minimizing false positives (FP). To create an ROC curve, TP rate is plotted against FP rate; the actual test statistic is the area under the curve (AUC) [24] (**Figure 5**). The larger AUC, the better the program's performance. The AUC for the unadjusted data was 0.854. ComBat_p and ComBat_n increased the AUC (0.937, p = 4.51e−30, p = 1.42e−29, respectively), followed by DWD (0.917, p = 5.88e−15), PAMR (0.913, p = 2.25e−13), and Ratio_G (0.895, p = 1.20e−06). SVA did not increase the AUC significantly (0.858, p = 0.27) (**Table S3, Row 15**). The results were similar in the Affymetrix spike-in data, except that SVA actually decreased the AUC value, from 0.93 to 0.76 (p<0.0001) (**Figure S4**).

To compare the methods' performances over a range of batch sizes, we re-set batch sizes at 20, 40 and 100 samples, re-generated the AAS data and recalculated the AUCs. The difference in AUC between ComBat and the other methods increased as the batch size decreased, suggesting that this Empirical Bayes approach is particularly appropriate for studies with fewer samples per batch (**Figure S5**).

## Discussion

In order to achieve adequate statistical power for population-based studies, sample sizes are considerably larger than the capacity of an individual microarray. Samples are unavoidably run in multiple batches due to technical and time constraints, making batch effects a source of non-biological variation that can increase error [25], [26]. When microarray technology was new, some researchers held that a well-designed experiment, using the same technician, lab and platform, could eliminate batch effects completely; as a result, many published reports of microarray data ignored batch effects in their analyses and even in their discussions. However, we now know that the causes of batch effects include variables simply not under the control of the researcher. Batch effects have been definitively demonstrated in microarray studies[7], [27], hence the development of algorithms designed to reduce them. In the SMRI brain expression microarray data set, batch effects accounted for nearly 50% of the observed variation in expression, to which site effects contributed 42% and date effects 7.3%.

We compared six methods for reducing or completely removing batch effects, using experimental and simulated microarray expression data. Although each method was effective according to one or more measures, ComBat outperformed other methods overall. Its parametric and non-parametric algorithms both worked well in both kinds of data sets, controlling the variation attributable to batch effects, increasing the correlation among replicates, and producing the largest AUC in our assessment of overall performance. We also confirmed another advantage of ComBat: it can robustly manage high-dimensional data when sample sizes are small, which is important for experiments with limited sample size, meta-analyses and clinical diagnostics. Moreover, ComBat not only worked well on data generated on the Affymetrix platform, but has also been reported to work well with Illumina BeadChips data [28].

The other commonly-used methods we evaluated were PAMR, DWD, Ratio_G and SVA. DWD did not perform well in our analyses when batch sizes were small. In addition, it can only analyze two batches at a time. Three or more batches may still be adjusted with DWD, using a stepwise approach[11], but this would not be convenient for large studies. Moreover, the standardization or normalization in DWD can change the scale between cases and controls, which is why the correlation between nominal and observed fold change was high after DWD processing, but the signal detection slope was very low. An addition re-scaling step would be required to allow results from different studies to be compared. SVA is based on SVD. It is effective, but has several limitations. First, it is not necessarily a simple matter to identify the batch effect eigenvector. Batch effects may actually contribute substantially to several of the top eigenvectors, so SVD may not identify and remove all the batch effects, and may remove other effects not related to batch. Second, a basic assumption of SVD is that the eigenvectors have Gaussian distributions. Batch effects, however, may be due to changes in technician, reagents, environmental conditions, scanner effects and/or other variables; this complicated situation may result in batch effects not being distributed in a Gaussian manner. Finally, SVD is not robust to outliers when compared with an Empirical Bayes method.

Ratio_G scales values by the geometric mean of a group of controls or reference samples, while PAMR shifts values by batches' arithmetic mean. Luo and colleagues [14] demonstrated that Ratio_G outperforms other methods in adjusting data for use in a predictive model, and reasoned that it is because non-ratio-based methods can confound batch and biological effects when one batch has a reverse negative/positive ratio compared to another batch. To test this, in our AAS data design, we simulated batches with very different case/control ratios, i.e., 10 cases and 30 controls in one batch and 30 cases and 10 controls in another. However, accuracy and ROC-AUC results indicated that Ratio_G performed worse than ComBat_p and ComBat_n. Also, Ratio_G performed worst in removing batch effects from the SMRI data.

PAMR sets the mean of each probe set within a given batch to zero. It did very well in our measures of accuracy because of this simple transformation. Again, though, batch effects are complicated, and do not affect all samples equally. PAMR does treat all samples equally, so it can over- or under-correct particular samples and came in second to ComBat in our measures of precision.

ComBat treats batch effects as additive and multiplicative effects. So it is basically a mixture of a mean-centering algorithm like PAMR, and a scale-based algorithm similar to Ratio G. This dual approach probably explains ComBat's superior overall performance.

Note, however, that even the most effective batch effect removal program cannot correct for poor experimental design. If cases and controls are run in separate batches, genuine biological variation can be entirely confounded by batch effects. Indeed, this is what we found when we re-configured our simulation data to run cases and controls separately, and re-analyzed it. No method was able to reduce the batch effects sufficiently without also removing the variation caused by case-control differences (**Table S2**).

A previous attempt to assess the extent of batch effects and the effectiveness of batch adjustment methods was made by the MAQC-II project [29]. This project's primary goal was to use existing data to create a model to predict class labels for future samples, where the classes, or endpoints, included treatment response, overall survival or likelihood of a specific disease. This predictive model is designed for use in both clinical settings and in research seeking to develop new gene expression signatures and biomarkers. Luo and colleagues investigated the effect of batch effect removal on the performance of this predictive model[14], as measured by Matthew Correlation Coefficient (MCC), using five different removal methods on six batches of MAQC microarray data, where sources of batch variation included different hybridization dates, different generations of chips, different channels, different platforms and different tissues.

They tested mean-centering, ratio-based, standardization and Empirical Bayes methods, and found them all to perform better than no batch adjustment 75% to 89% of the time, with the geometric ratio-based (Ratio_G) method performing the best. DWD and SVD/SVA were not evaluated, because Luo and colleagues wanted to develop their predictive model based on the existing data, referred to as the training data, which would not be affected when the model was applied to future samples. They used MCC because it is informative with very different class sizes, straightforward to calculate, and applicable for all 30,000+ of their models.

We, on the other hand, assessed how batch adjustment methods improve the integration of data processed at different sites or times when used as a standard quality control step post-RMA and pre-integration. This meant not only that we had a different aim than Luo and colleagues, but that we had fewer constraints. For example, we were able to assess widely-used algorithms, such as DWD and SVD/SVA, which they did not since would alter the training data. Also, although when we calculated MCC for our data (**Table S3, Row 9**) the results mirrored our accuracy and ROC-AUC results, the precision results elucidated differences among the programs not revealed by MCC, our accuracy measures or the ROC curves. For example, by testing multiple aspects of performance, rather than relying on a single measure, we demonstrated that while SVA substantially reduced the proportion of variation attributable to batch effects, as well performing with precision, it actually decreased accuracy relative to the unadjusted data. We also showed that PAMR performed very accurately, but slightly less precisely. We performed each test on both simulation data and experimental data; the simulation data let us control the variables and know the true values a *priori*, and then we were able to validate the simulation results in real data. Because we were not creating a prediction model, which is a combination analysis with batch correction, feature selection, classification and model selection, we were able to assess data before and after batch adjustment directly: this eliminates the potential for variation being introduced by those additional procedures. Finally, we took only date and site effects into consideration, since platform-, channel- or tissue-dependent variations are avoidable with careful experimental design.

These differences in research design can explain why Luo and colleagues rated Ratio_G the highest, while we found ComBat to be superior. For example, Ratio G performed particularly well only in one MAQC data set, a data set for which the stated source of batch effect was cross-tissue. However, cross-tissue effects would be expected to be due to biological variation between tissues as well as technical variation, presumably related to tissue collection and extraction. So, it is not clear whether all the variation removed was technical or whether all the technical variation was removed. Moreover, in the predicted model summary, this data set was used in 32 of 120 models, while other data sets were only used in 8 or 16 models, leading to Ratio_G's superior performance in the MAQC analysis. If we examine separately the MAQC-II data sets in which hybridization date is the only source of batch effects, ComBat appears to increase MCC at least as well as Ratio_G does [14].

We detected a strong probe level effect after applying RMA to the SMRI data and finding that the probe set expression values among replicates correlated no better than among non-replicates. We observed that the array level variation (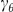) was well-controlled, but the probe set level variation (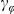) was not alleviated by RMA (**Figure S6**), which is consistent with a previous study[23]. As the correlation between each individual array is 
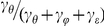
 where 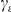 represents the variation of measurement error, a high 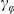 will result in an artificially high correlation between different arrays. To solve this, we standardized the probe set variation by standardizing means and variances across replicate groups, which keeps the variability between pairs but lowers the overall variation. After standardization, correlation among non-replicates fell to zero (**Figure S1**).

Our evaluation makes clear that adjustment for batch effects is a mandatory step in the analysis of microarray data when the sample size is too large to fit in a single batch. Of the batch adjustment methods we evaluated, we found that the empirical Bayes algorithm implemented in ComBat was best able to reduce and remove batch effects while increasing precision and accuracy. It outperformed the second best program by a substantial margin on many measures and by a small but significant margin on others. Unlike the other programs, each of which had at least one major drawback, ComBat performed satisfactorily on all measures. PAMR was a close second, but its performance suffered when batch size was small; only ComBat performed robustly when adjusting small batches. Finally, we recommend that probe set expression values always be standardized prior to assessing correlation among replicates, to avoid misleadingly high correlations.

## Materials and Methods

### Samples

The SMRI brain samples came from two brain collections, the SMRI Array collection and the SMRI Consortium collection[18]. The SMRI Array collection includes 70 patients and 35 controls. The Brodmann area 46 of each sample was assayed in three groups using the Affymetrix GeneChip Human Genome U133A platform. The three studies had 62 samples in common after outliers were removed using Expression Console[30], which we used as our technical replicates. The 7643 probes that were coded as present call in 80% or more of the samples were included in our analyses[31].

### Expression microarray data simulations

We generated two simulated expression microarray data sets. In the variation assessment simulated (VAS) data, we generated 100 samples for which 1000 probes were measured. Samples were assigned to one of two undefined profiles, which could represent disease status, drug treatment or similar random variable, 65 to Profile 1 and 35 to Profile 2. The simulation was run twice: the first replicate group was generated as if run in a single batch, while the second replicate group generated as if run in two batches. To address batch effects and treatment effect, we assumed the data followed an L/S model,



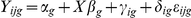
 (1),

Where 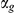 represents the overall gene expression, *X* is a design matrix for profile conditions, and 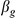 is the vector of regression coefficients corresponding to *X*. The additive and multiplicative batch effects of batch *i* for gene *g* are represented by 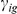 and 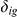
_,_ respectively. Measurement error is represented by
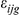
_,_ and is normally distributed, with an expected value of zero and variance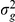.

Parameters 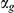 and 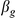 were set such that probe sets were differentially expressed between the two profiles. Across Profile 1, probe sets' average expression intensities followed a normal distribution, and across Profile 2, probe sets' expression intensities have a variety of fold changes relative to Profile 1, ranging from −3 to 3. Standard deviation of gene g was set to ensure that the gene was genuinely differently expressed, rather than highly variable: 
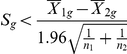
, where 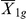 is the mean intensity of gene g and 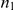 is the sample size of Profile 1, and 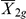 is the mean intensity and 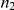 is the sample size of Profile 2. After significance testing and multiple test correction, we were left with 991 differentially expressed probe sets with various fold changes for further analysis. Batch scale parameters,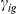, 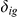, were added to reflect a typical range of observed values, which followed normal distribution and inverse-gamma distribution, respectively. All the distribution and fold change parameters were set to reflect those of the biological data.

The second simulated data set was the accuracy assessment simulated (AAS) data, which included probe set expression values for 10,000 probe sets in 100 controls and 100 patients. We again followed Equation (1), where *X* is a design matrix for sample conditions. Parameters 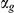 and 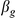 were set such that 1200 of the 10,000 genes had mean fold changes ranging from −3 to 3 in cases relative to controls. Approximate standard deviation was set so that some lower fold change genes were differentially expressed, so we could test whether small fold changes were still detectable after batch correction.

### Measuring source of variation

The PVCA approach (http://www.niehs.nih.gov/research/resources/software/pvca/index.cfm) was used to estimate sources of variability and compare batch effects before and after adjustment in the VAS and SMRI microarray data sets. We first selected the top PCs, enough to explain a proportion of overall variation larger than a pre-defined threshold (60%–90%, 60% in this case), and retained the corresponding eigenvalues. Each factor was treated as random in a mixed linear model and matched to a PC, then weighted by that PC's corresponding eigenvector. After we standardized the variation attributable to each factor, we calculated the proportion of total variance each factor explained. Including residuals, four factors' variations were estimated in the VAS data and ten factors' variations were estimated in SMRI data.

Signal detection slopes were calculated using the spkTools[21] R package. The significances of differences between slopes were assessed with a test for homogeneity of slope[32], which was done with the NCStats R package[33]. The evaluation of overall performance was performed using the ROCR[34] R package from Bioconductor[35].

## Supporting Information

Figure S1 **Correlation before and after standardization.** Compare the replicate samples correlation between pre-standardization and post-standardization. Data was downloaded from Affymetrix U133A sample data including three replicates for 12 different tissues. Affy12, affy23, affy13 are replicates' correlation groups between replicate group 1 and replicate group 2, replicate group 2 and replicate group 3, replicate group 1 and replicate group 3, respectively. AffyN12, affyN23, affyN13 are non-replicates' correlation between group 1 and 2, group 2 and 3, group 1 and 3, respectively. http://www.affymetrix.com/support/technical/sample_data/gene_1_0_array_data.affx.
(TIF)

Figure S2 **Distribution of z scores in VAS data.** Box plots of the distribution of z scores transformed from Pearson correlation coefficients between simulated replicates. The methods are listed along the X axis. The Y axis is the distributions of all probes' z scores. The top of the box represents top of the third quartile, the bottom of the box represents the bottom of the first quartile, the middle bar is the median value, box whiskers extend to 1.5 times the interquartile range from the box and circles are possible outliers. The differences of correlation distribution are all significant with p value less than 0.0001; differences of distribution's median between RAW data and data have been processed with batch-adjustment methods are listed below the box plots.
(TIF)

Figure S3 **Slope test in Affymetrix spike-in data.** Observed versus nominal values in Affymetrix Latin square design spike in data, for RMA data and post batch adjustment methods. Expression values are plotted against the log (base 2) of the reported nominal concentration. The regression slope obtained utilizing all the data and the regression slopes obtain within each low, medium and high average log expression (ALE) value strata are shown. The slope of each line is reported in the legend. The vertical lines divide the ALE strata.
(TIFF)

Figure S4 **ROC curves in Affymetrix spike-in data.** ROC curves are graphical representations of both specificity and sensitivity that take into account both differentially and non-differentially expressed genes. Concentration pairs with fold-changes of 2 in spike-in genes were used to determine TP. Concentration pairs without any fold-changes were used to determine TN. We selected top 1000 log-ratio pairs to report.
(TIFF)

Figure S5 **Compare of the AUCs in AAS data.** The differences of the AUC between ComBat and the next best batch adjusted method are different when the sample size of each batch varies. From the left, the differences are 0.03, 0.02 and 0.01, as the batch sizes increase from 20, 40 to 100.
(TIFF)

Figure S6 **Array level and probe set level variation.** Boxplot of the RMA normalized SMRI data for (A) all the 186 arrays and (B) all 7643 probe sets. Y axis is the log2 intensity value and x axis is the (A) arrays or (B) probe sets. After RMA, gene intensity distributions are similar between arrays but not probe sets, leading to artificially large correlations between non-replicate arrays.
(TIFF)

Table S1 **Detailed computational description of six programs.** Detailed computational features of each program are provided, including software implement, file format, relative execution time, computational burden, batch size, URL of the available software and some program-specific notes.
(DOC)

Table S2 **Batch effect completely confounded with outcome variation.** We simulated the data with all cases in one batch and all controls in another. There are 1200 true positive genes in this dataset. The binary classification table shows overlapped positives genes among the true condition and with or without batch adjustment methods: before batch correction, there were 6518 significant results in raw data, with 62.4% (5495 out of 8800) false positive rate (FPR) and 14.8% (177 out of 1200) false negative rate (FNR); After batch correction, ComBat_p got 7 positive results with 0% FPR and 99.0% FNR; ComBat_n got 12 positive results with 0.1% FPR and 99.8% FNR; DWD got 402 positive results with 3.2% FPR and 89.9% FNR. None of the true positives were caught by PAMR and SVA with 0% FPR and 100% FNR. No control samples in the first batch so Ratio_G can't be applied for this adjustment.
(DOC)

Table S3 **Assessment summary statistics table.** The second column indicates the figure to which the summary statistic relates. Columns 3 through 9 show values for RMA, ComBat_p, ComBat_n, PAMR, DWD, SVA and Ratio_G, separately. The statistics are described in the text and best result is shown in bold. Abbreviations: PVCA, principal variation component analysis; ICC, intraclass correlation; ACC, accuracy; MCC, Matthew Correlation Coefficient; AUC, area under the curve.
(DOC)
