# Supplementary figures and images for: Removing Batch Effects in Analysis of Expression Microarray Data: An Evaluation of Six Batch Adjustment Methods
Source: PLoS One. 2011 Feb 28;6(2):e17238. doi: 10.1371/journal.pone.0017238 (PMC3046121; doi:10.1371/journal.pone.0017238)

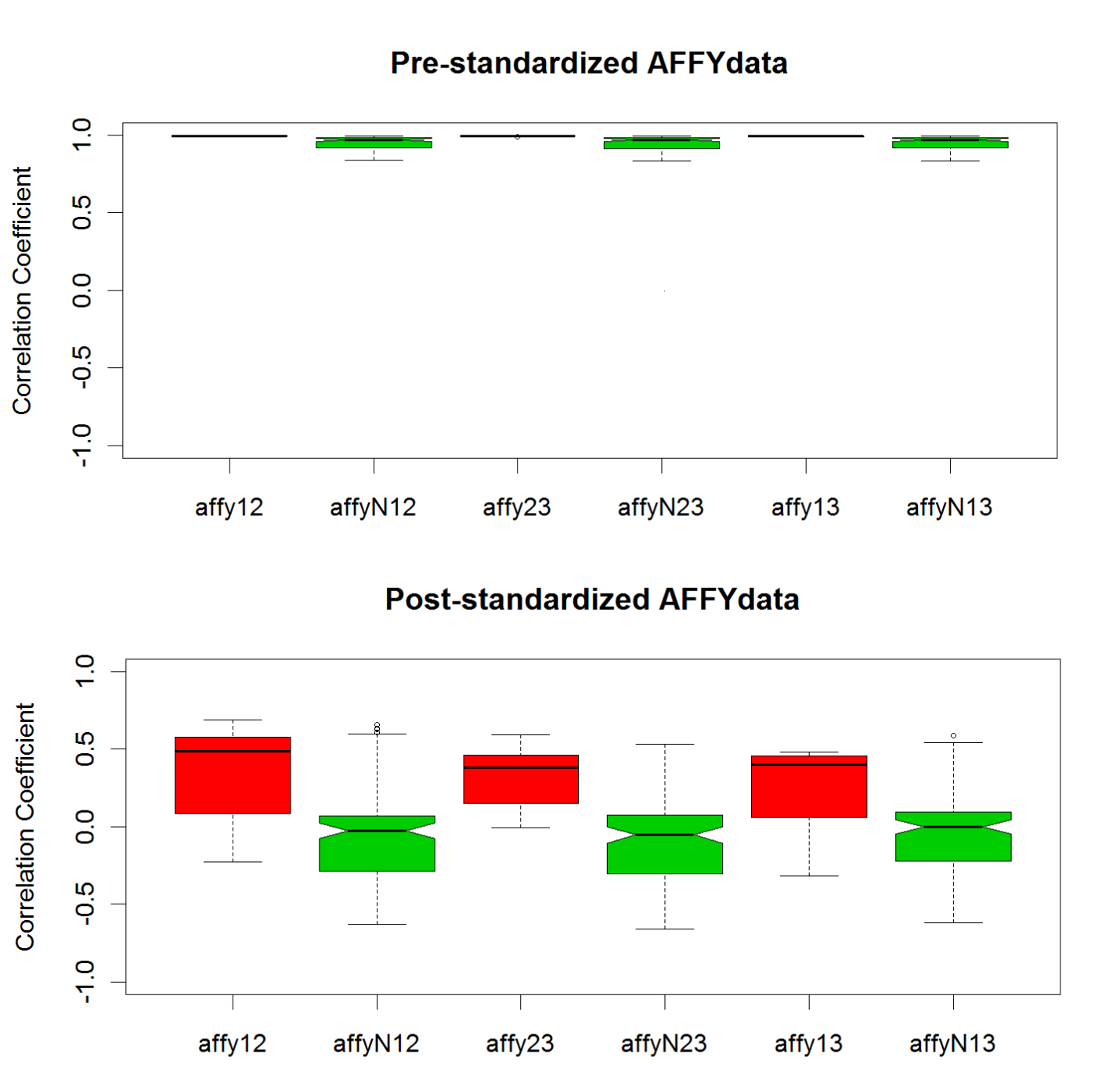

Supplement: Figure S1 — Correlation before and after standardization. Compare the replicate samples correlation between pre-standardization and post-standardization. Data was downloaded from Affymetrix U133A sample data including three replicates for 12 different tissues. Affy12, affy23, affy13 are replicates' correlation groups between replicate group 1 and replicate group 2, replicate group 2 and replicate group 3, replicate group 1 and replicate group 3, respectively. AffyN12, affyN23, affyN13 are non-replicates' correlation between group 1 and 2, group 2 and 3, group 1 and 3, respectively. http://www.affymetrix.com/support/technical/sample_data/gene_1_0_array_data.affx. (TIF) [file pone.0017238.s001.tif]

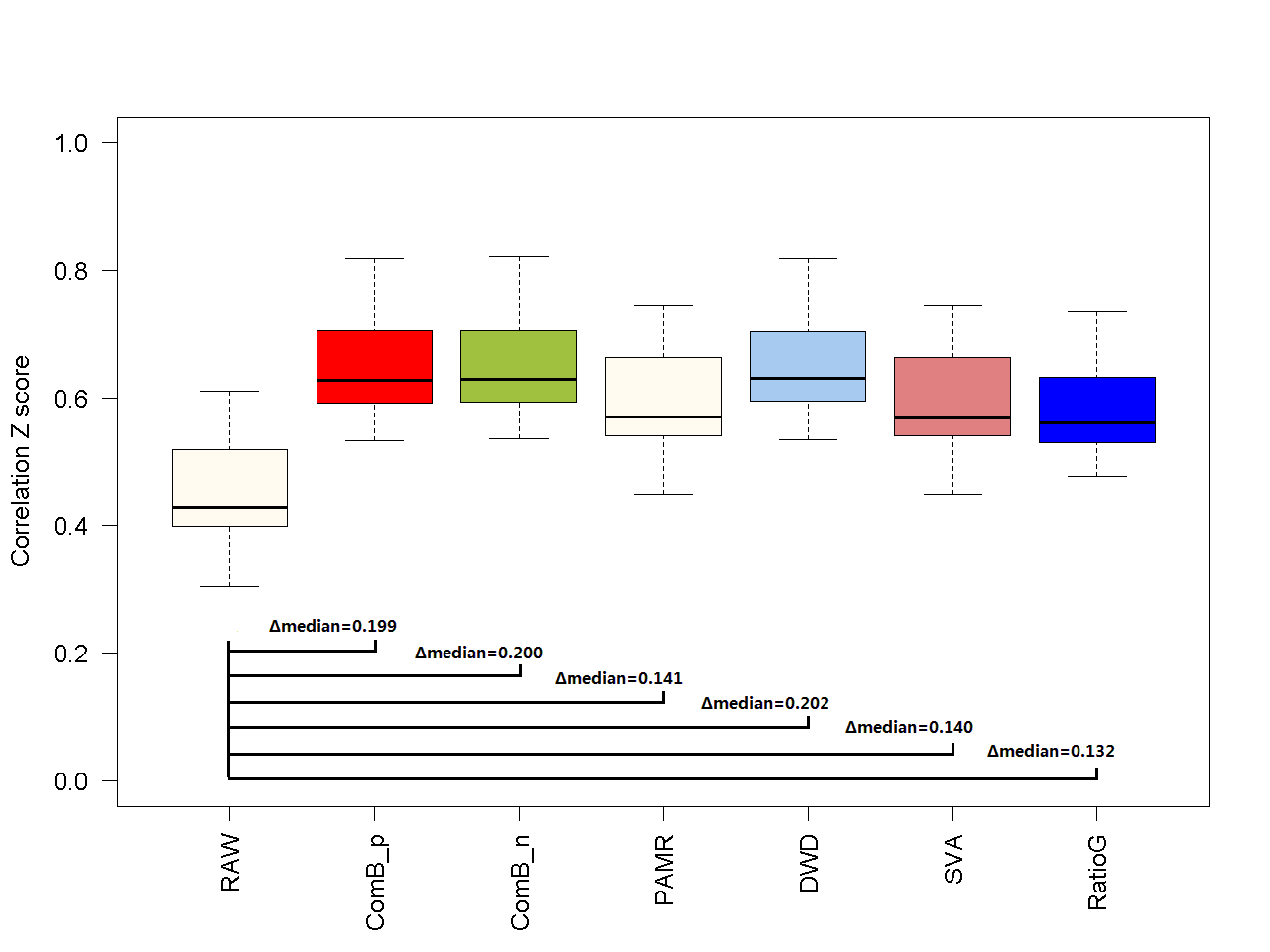

Supplement: Figure S2 — Distribution of z scores in VAS data. Box plots of the distribution of z scores transformed from Pearson correlation coefficients between simulated replicates. The methods are listed along the X axis. The Y axis is the distributions of all probes' z scores. The top of the box represents top of the third quartile, the bottom of the box represents the bottom of the first quartile, the middle bar is the median value, box whiskers extend to 1.5 times the interquartile range from the box and circles are possible outliers. The differences of correlation distribution are all significant with p value less than 0.0001; differences of distribution's median between RAW data and data have been processed with batch-adjustment methods are listed below the box plots. (TIF) [file pone.0017238.s002.tif]

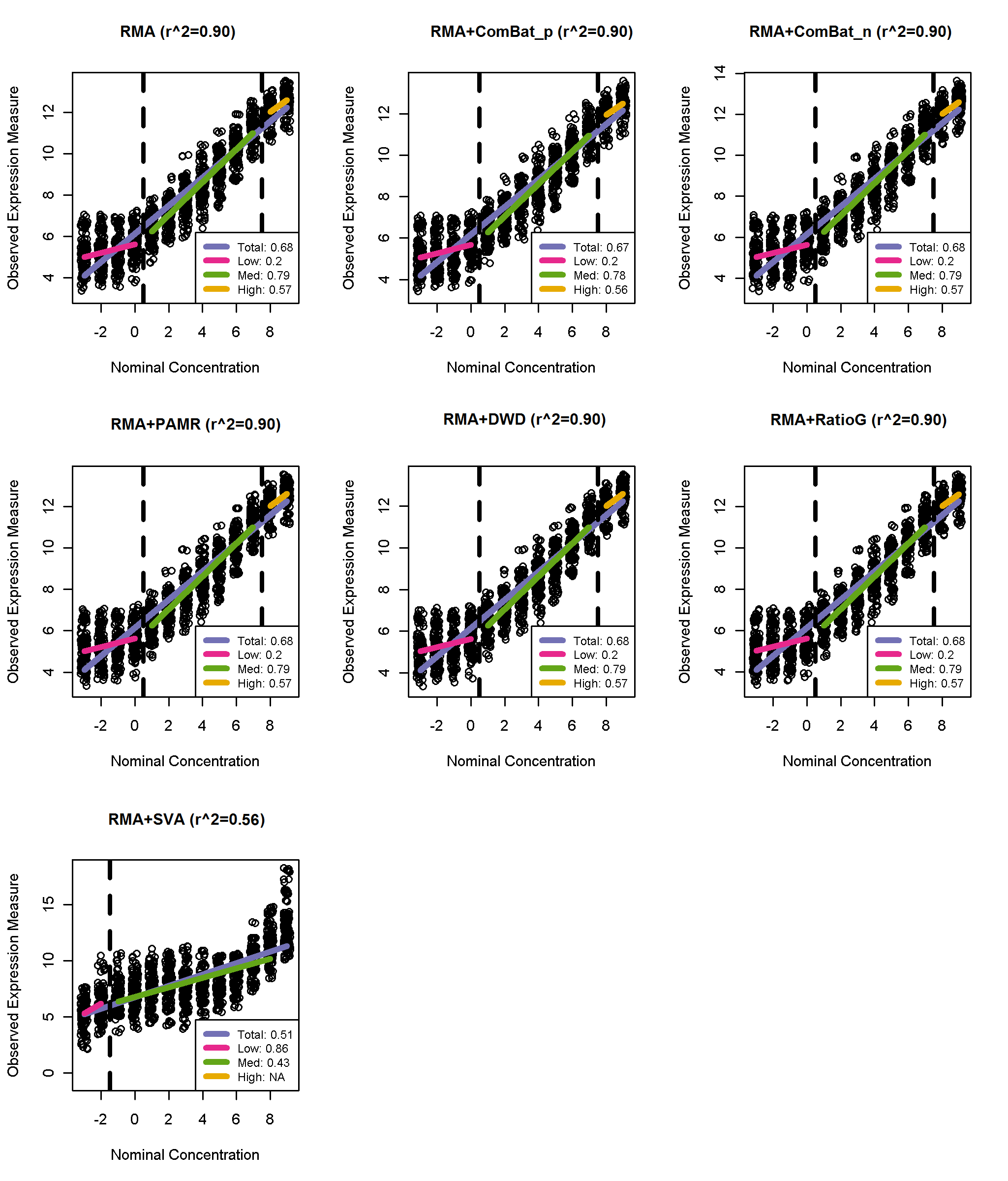

Supplement: Figure S3 — Slope test in Affymetrix spike-in data. Observed versus nominal values in Affymetrix Latin square design spike in data, for RMA data and post batch adjustment methods. Expression values are plotted against the log (base 2) of the reported nominal concentration. The regression slope obtained utilizing all the data and the regression slopes obtain within each low, medium and high average log expression (ALE) value strata are shown. The slope of each line is reported in the legend. The vertical lines divide the ALE strata. (TIFF) [file pone.0017238.s003.tiff]

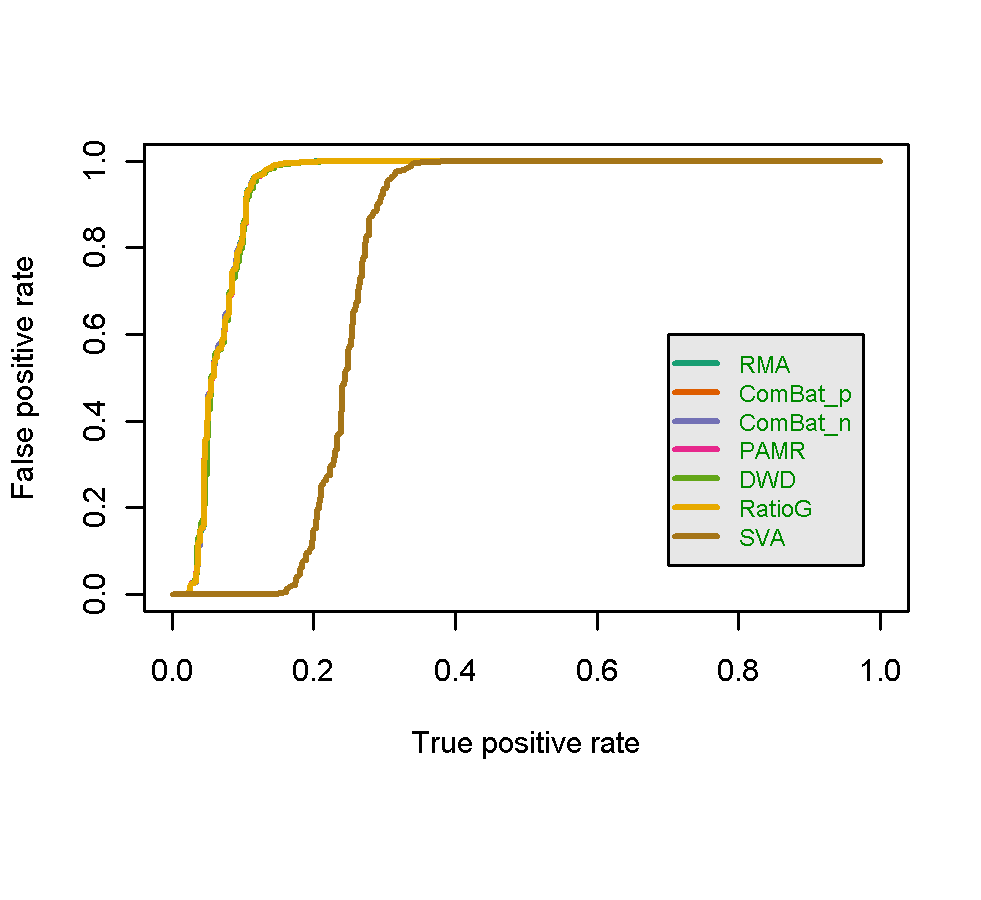

Supplement: Figure S4 — ROC curves in Affymetrix spike-in data. ROC curves are graphical representations of both specificity and sensitivity that take into account both differentially and non-differentially expressed genes. Concentration pairs with fold-changes of 2 in spike-in genes were used to determine TP. Concentration pairs without any fold-changes were used to determine TN. We selected top 1000 log-ratio pairs to report. (TIFF) [file pone.0017238.s004.tiff]

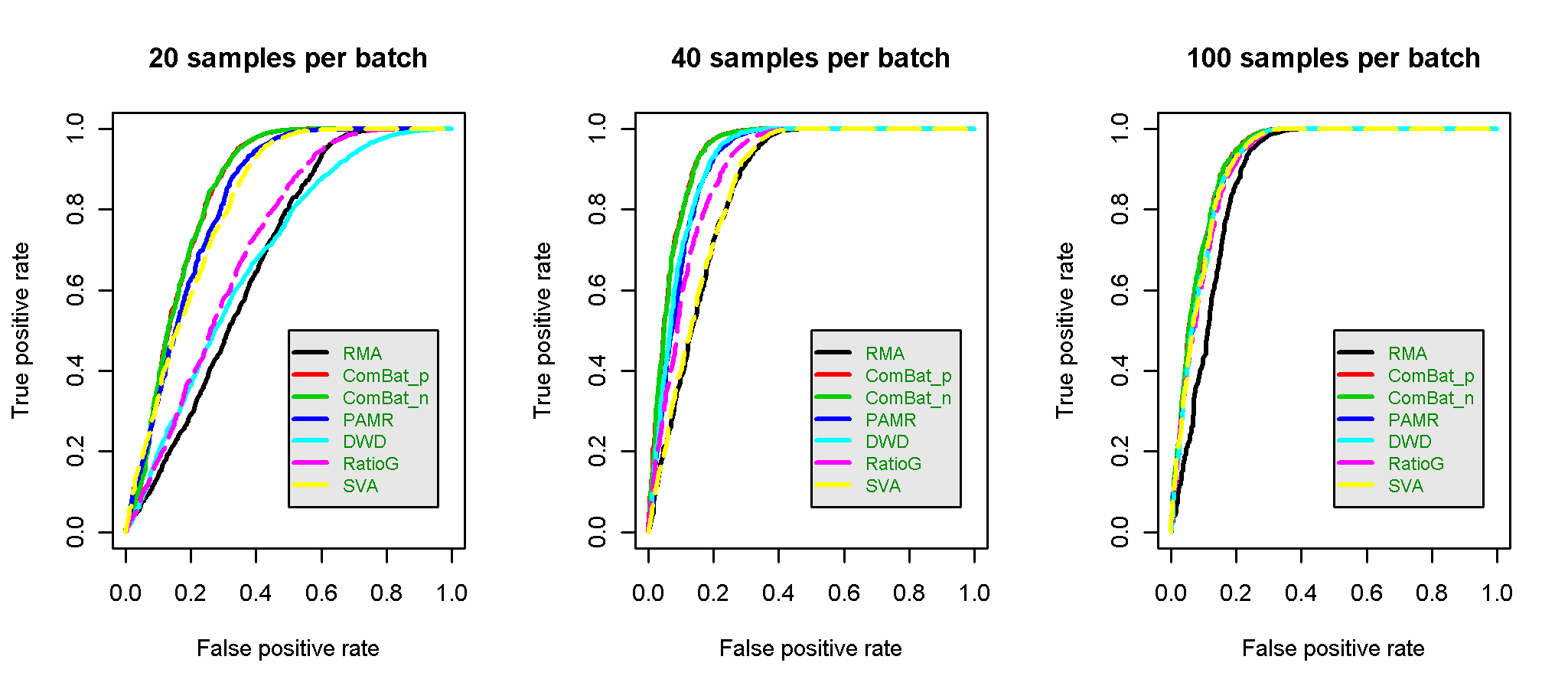

Supplement: Figure S5 — Compare of the AUCs in AAS data. The differences of the AUC between ComBat and the next best batch adjusted method are different when the sample size of each batch varies. From the left, the differences are 0.03, 0.02 and 0.01, as the batch sizes increase from 20, 40 to 100. (TIFF) [file pone.0017238.s005.tiff]

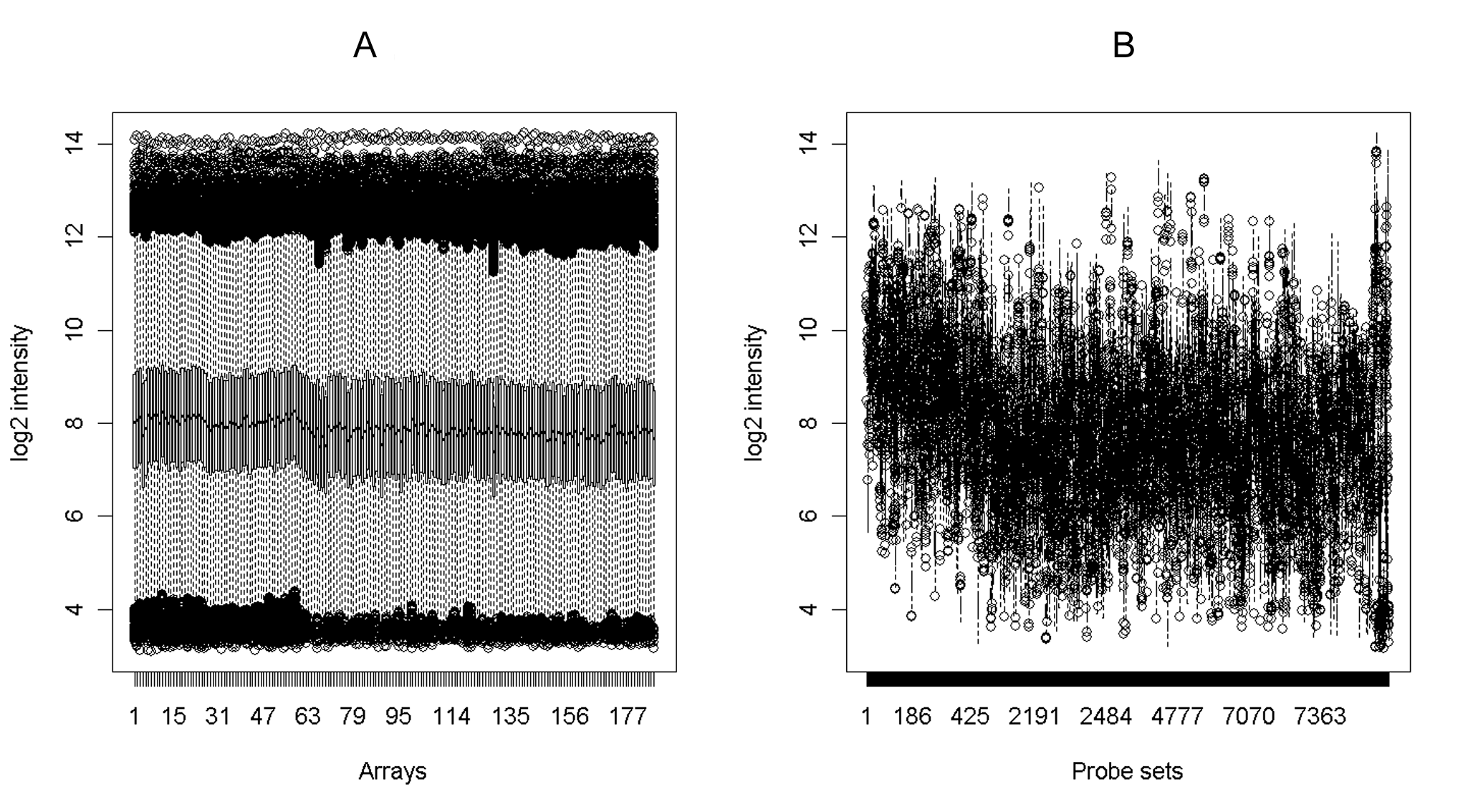

Supplement: Figure S6 — Array level and probe set level variation. Boxplot of the RMA normalized SMRI data for (A) all the 186 arrays and (B) all 7643 probe sets. Y axis is the log2 intensity value and x axis is the (A) arrays or (B) probe sets. After RMA, gene intensity distributions are similar between arrays but not probe sets, leading to artificially large correlations between non-replicate arrays. (TIFF) [file pone.0017238.s006.tif]
