# Supplementary material for: Removing Batch Effects in Analysis of Expression Microarray Data: An Evaluation of Six Batch Adjustment Methods
Source: PLoS One. 2011 Feb 28;6(2):e17238. doi: 10.1371/journal.pone.0017238 (PMC3046121; doi:10.1371/journal.pone.0017238)
Supplement: Table S1 — Detailed computational description of six programs. Detailed computational features of each program are provided, including software implement, file format, relative execution time, computational burden, batch size, URL of the available software and some program-specific notes. (DOC) [file pone.0017238.s007.doc]

Table S1. Batch effect completely confounded with outcome variation

|  |  | True Positives(1200) | True Negatives(8800) |
| --- | --- | --- | --- |
| Raw data | Observed Positives | 1023 | 5495 |
|  | Observed Negatives | 177 | 3305 |
| ComBat_p | Observed Positives | 12 | 0 |
|  | Observed Negatives | 1188 | 8800 |
| ComBat_n | Observed Positives | 2 | 5 |
|  | Observed Negatives | 1198 | 8795 |
| PAMR | Observed Positives | 0 | 0 |
|  | Observed Negatives | 1200 | 8800 |
| DWD | Observed Positives | 121 | 281 |
|  | Observed Negatives | 1079 | 8519 |
| SVA | Observed Positives | 0 | 0 |
|  | Observed Negatives | 1200 | 8800 |

We simulated the data with all cases in one batch and all controls in another. There are 1200 true positive genes in this dataset. The [binary classification](http://en.wikipedia.org/wiki/Binary_classification) table shows overlapped positives genes among the true condition and with or without batch adjustment methods: before batch correction, there were 6518 significant results in raw data, with 62.4% (5495 out of 8800) false positive rate (FPR) and 14.8% (177 out of 1200) false negative rate (FNR); After batch correction, ComBat_p got 7 positive results with 0% FPR and 99.0% FNR; ComBat_n got 12 positive results with 0.1% FPR and 99.8% FNR; DWD got 402 positive results with 3.2% FPR and 89.9% FNR. None of the true positives were caught by PAMR and SVA with 0% FPR and 100% FNR. No control samples in the first batch so Ratio_G can’t be applied for this adjustment.
