# Supplementary material for: Removing Batch Effects in Analysis of Expression Microarray Data: An Evaluation of Six Batch Adjustment Methods
Source: PLoS One. 2011 Feb 28;6(2):e17238. doi: 10.1371/journal.pone.0017238 (PMC3046121; doi:10.1371/journal.pone.0017238)
Supplement: Table S2 — Batch effect completely confounded with outcome variation. We simulated the data with all cases in one batch and all controls in another. There are 1200 true positive genes in this dataset. The binary classification table shows overlapped positives genes among the true condition and with or without batch adjustment methods: before batch correction, there were 6518 significant results in raw data, with 62.4% (5495 out of 8800) false positive rate (FPR) and 14.8% (177 out of 1200) false negative rate (FNR); After batch correction, ComBat_p got 7 positive results with 0% FPR and 99.0% FNR; ComBat_n got 12 positive results with 0.1% FPR and 99.8% FNR; DWD got 402 positive results with 3.2% FPR and 89.9% FNR. None of the true positives were caught by PAMR and SVA with 0% FPR and 100% FNR. No control samples in the first batch so Ratio_G can't be applied for this adjustment. (DOC) [file pone.0017238.s008.doc]

Table S2. Assessment summary statistics table

| Assessment | metrics | RMA | ComBat_p | ComBat_n | PAMR | DWD | SVA | Ratio_G |
| --- | --- | --- | --- | --- | --- | --- | --- | --- |
| Source of variance (PVCA on VAS data, % of variation attributed to batch effect) | 1 | 30.4% | **0%** | **0%** | **0%** | 2.9% | 0.1% | 3.8% |
| Source of variance (Date effect on SMRI data) | 2 | 7.3% | **0%** | **0%** | 1% | 11% | 3% | 10% |
| Source of variance (Site effect on SMRI data) | 3 | 42.2% | 1% | 1% | **0%** | 2% | **0%** | 11% |
| Δmedian of correlation R transformed z-score (VAS data) | 4 | - | **0.20** | **0.20** | 0.14 | **0.20** | 0.14 | 0.13 |
| Δmedian of ICC R transformed z-score (SMRI data) | 5 | - | 0.12 | 0.12 | 0.11 | **0.13** | 0.02 | 0.08 |
| Significance of difference between RMA median z and batch-adjusted median z distribution (VAS data) | 6 | - | **p<0.0001** | **p<0.0001** | **p<0.0001** | **p<0.0001** | **p<0.0001** | **p<0.0001** |
| Significance of difference between RMA median z and batch-adjusted median z distribution (SMRI data) | 7 | - | **p<0.0001** | **p<0.0001** | **p<0.0001** | **p<0.0001** | p=0.407 | **p<0.0001** |
| ACC (AAS data) | 8 | 0.79 | **0.96** | **0.96** | 0.94 | 0.94 | 0.79 | 0.91 |
| MCC (AAS data) | 9 | 0.41 | **0.80** | 0.79 | 0.72 | 0.71 | 0.41 | 0.63 |
| Signal detection slope (AAS data) | 10 | 1.01 | **0.99** | **0.99** | **0.99** | 0.94 | 0.94 | 0.90 |
| Signal detection slope (Affy spike-in data) | 11 | **0.68** | 0.67 | **0.68** | **0.68** | **0.68** | 0.51 | **0.68** |
| Correlation between nominal and observed FCs (AAS data) | 12 | 0.95 | **0.98** | **0.98** | 0.97 | 0.97 | 0.95 | 0.96 |
| Correlation between nominal and observed FCs (Affy spike in data) | 13 | **0.90** | **0.90** | **0.90** | **0.90** | **0.90** | 0.56 | **0.90** |
| AUC, batch size=100 (AAS data) | 14 | 0.88 | **0.93** | **0.93** | 0.92 | 0.92 | 0.92 | 0.92 |
| AUC, batch size=40 (AAS data) | 15 | 0.85 | **0.94** | **0.94** | 0.91 | 0.92 | 0.86 | 0.90 |
| AUC, batch size=20 (AAS data) | 16 | 0.68 | **0.85** | **0.85** | 0.82 | 0.67 | 0.82 | 0.72 |
| AUC (Affy spike in data) | 17 | **0.93** | **0.93** | **0.93** | **0.93** | **0.93** | 0.76 | **0.93** |

The second column indicates the figure to which the summary statistic relates. Columns 3 through 9 show values for RMA, ComBat_p, ComBat_n, PAMR, DWD, SVA and Ratio_G. The statistics are described in the text and best result is shown in bold. Abbreviations: PVCA, principal variation component analysis; ICC, intraclass correlation; ACC, accuracy; MCC, Matthew Correlation Coefficient; AUC, area under the curve.
