# Supplementary material for: Removing Batch Effects in Analysis of Expression Microarray Data: An Evaluation of Six Batch Adjustment Methods
Source: PLoS One. 2011 Feb 28;6(2):e17238. doi: 10.1371/journal.pone.0017238 (PMC3046121; doi:10.1371/journal.pone.0017238)
Supplement: Table S3 — Assessment summary statistics table. The second column indicates the figure to which the summary statistic relates. Columns 3 through 9 show values for RMA, ComBat_p, ComBat_n, PAMR, DWD, SVA and Ratio_G, separately. The statistics are described in the text and best result is shown in bold. Abbreviations: PVCA, principal variation component analysis; ICC, intraclass correlation; ACC, accuracy; MCC, Matthew Correlation Coefficient; AUC, area under the curve. (DOC) [file pone.0017238.s009.doc]

| **Program** | **Software implement** | **File format**  **requirement** | **Relative**  **execution time** | **Computational burden**  **(memory limit)** | **Batch size** | **Note** | **URL** |
| --- | --- | --- | --- | --- | --- | --- | --- |
| ComBat_p | R | Expression file: tab-delimited .txt file with sample information in columns and genes in rows  Sample info file:  Tab-delimited .txt file with column name (Array, Sample, Batch and Covariate). Covariate is optional and must be a categorical factor | Medium | 32-bit Windows: The address-space limit is up to 3Gb; 64-bit Windows: 1) 32-bit **R:** limit is 2Gb for oldest 64-bit Windows; other 64-bit versions, limit is 4Gb, 2) 64-bit R**:** limit is 8Tb  Unix: 1) 32-bit R on 32-bit or 64-bit OS imposes a limit of 4Gb; 2) 64-bit R has a 128Tb limit for Linux on x86_64 CPUs | Performs well with both big and small batches | Forum <http://groups.google.com/group/combat-user-forum/?pli=1>  Expression data can be exported from dChip software | http://jlab.byu.edu//ComBat/Usage.html |
| ComBat_n | R | Same as ComBat_p | Slow (needs more time to estimate parameters because no prior distributions) | Memory limitation issue: Same as ComBat_p | Performs well with both big and small batches | Forum  <http://groups.google.com/group/combat-user-forum/?pli=1>  Expression data can be exported from dChip software | http://jlab.byu.edu//ComBat/Usage.html |
| PAMR | R | Expression file: tab-delimited .txt file or comma-delimited .csv file with sample information in columns and genes in rows  A vector of the class labels for each sample, and *batchlabels,* a vector of batch labels for each sample, are needed. | Fast | Memory limitation issue: Same as ComBat_p | Not robust when batch sizes are small | Function *pamr. batchadjust* in PAMR R package | http://www.bioconductor.org/help/bioc-views/2.8/bioc/html/pamr.html |
| DWD | JAVA | Different batches must be splitted to separate files | Slow (needs additional step to split the batch files) | Only two batches can be analyzed at a time. Stepwise approach is not convenient for large studies with more batches.  Also there is a similar memory limitation as ComBat_p. | Not robust when batch sizes are small |  | <https://genome.unc.edu/pubsup/dwd/> |
| SVA | R | Expression file: tab-delimited .txt file or comma-delimited .csv file with sample information in columns and genes in rows  For differential expression analysis, group information is needed. | Slow | Memory limitation issue: same as ComBat_p | Not robust when batch sizes are small | SVA/SVD may not identify and remove all the batch effects, if batch effects contribute substantially to the top eigenvectors. | http://www.biostat.jhsph.edu/~jleek/sva/ |
| Ratio_G | R | Expression file: tab-delimited .txt file or comma-delimited .csv file with sample information in columns and genes in rows  Normal samples are needed as reference measurements. | Fast | Memory limitation issue: same as ComBat_p | Not robust when batch sizes are small | Function *geometric.mean* in Psych R package | http://cran.r-project.org/web/packages/psych/index.html |
